# Evaluating the Prognostic and Clinical Validity of the Fall Risk Score Derived From an AI-Based mHealth App for Fall Prevention: Retrospective Real-World Data Analysis

**DOI:** 10.2196/55681

**Published:** 2024-12-04

**Authors:** Sónia A Alves, Steffen Temme, Seyedamirhosein Motamedi, Marie Kura, Sebastian Weber, Johannes Zeichen, Wolfgang Pommer, André Baumgart

**Affiliations:** 1 LINDERA GmbH, Modersohnstraße 36, Berlin, 10245, Germany, 49 030 12085471; 2 Johannes Wesling Klinikum Minden - Klinik für Unfallchirurgie und Orthopädie, Minden, Germany; 3 Charité - Universitätsmedizin Berlin, Berlin, Germany; 4 Department of Anesthesiology and Surgical Intensive Care Medicine, Medical Faculty Mannheim, University Medical Center GmbH, Heidelberg University, Mannheim, Germany; 5 Medical Faculty Mannheim, Department of Biomedical Informatics, University Medical Centre Mannheim GmbH, Heidelberg University, Mannheim, Germany

**Keywords:** falls, older adults, mHealth, prognostic tool, clinical validity, AI, mobile health, artificial intelligence

## Abstract

**Background:**

Falls pose a significant public health concern, with increasing occurrence due to the aging population, and they are associated with high mortality rates and risks such as multimorbidity and frailty. Falls not only lead to physical injuries but also have detrimental psychological and social consequences, negatively impacting quality of life. Identifying individuals at high risk for falls is crucial, particularly for those aged ≥60 years and living in residential care settings; current professional guidelines favor personalized, multifactorial fall risk assessment approaches for effective fall prevention.

**Objective:**

This study aimed to explore the prognostic validity of the Fall Risk Score (FRS), a multifactorial-based metric to assess fall risk (using longitudinal real-world data), and establish the clinical relevance of the FRS by identifying threshold values and the minimum clinically important differences.

**Methods:**

This retrospective cohort study involved 617 older adults (857 observations: 615 of women, 242 of men; mean age 83.3, SD 8.7 years; mean gait speed 0.49, SD 0.19 m/s; 622 using walking aids) residing in German residential care facilities and used the LINDERA mobile health app for fall risk assessment. The study focused on the association between FRS at the initial assessment (T1) and the normalized number of falls at follow-up (T2). A quadratic regression model and Spearman correlation analysis were utilized to analyze the data, supported by descriptive statistics and subgroup analyses.

**Results:**

The quadratic model exhibited the lowest root mean square error (0.015), and Spearman correlation analysis revealed that a higher FRS at T1 was linked to an increased number of falls at T2 (ρ=0.960, *P*<.001). Subgroups revealed significant strong correlations between FRS at T1 and falls at T2, particularly for older adults with slower gait speeds (ρ=0.954, *P*<.001) and those using walking aids (ρ=0.955, *P*<.001). Threshold values revealed that an FRS of 45%, 32%, and 24% corresponded to the expectation of a fall within 6, 12, and 24 months, respectively. Distribution-based minimum clinically important difference values were established, providing ranges for small, medium, and large effect sizes for FRS changes.

**Conclusions:**

The FRS exhibits good prognostic validity for predicting future falls, particularly in specific subgroups. The findings support a stratified fall risk assessment approach and emphasize the significance of early and personalized intervention. This study contributes to the knowledge base on fall risk, despite limitations such as demographic focus and potential assessment interval variability.

## Introduction

Falls represent a major health risk, with a profound impact on both individuals and society, particularly for those aged ≥60 years [1]. They are inherently linked to adverse effects on mobility, concurrent care risks, increased disease burden, and increased mortality rates [2-5]. Falls are the second leading cause of unintentional injury deaths globally, with an estimated 684,000 fatalities annually [6]. The number of falls and their related injuries are estimated to likely increase in the upcoming years [7]. This occurrence can be attributed in part to increasing life expectancy [8] and a rising prevalence of fall risk factors, including but not limited to multimorbidity, polypharmacy, and frailty [7]. Fall-related injuries often lead to severe consequences [9], including hip fractures [10] and head trauma [11], which can increase the risk of death, disability, and institutional care, as well as impose substantial economic strain on the health care system [12]. Falls can also have a significant impact on quality of life. Fear of falling can lead to social isolation, reduced physical activity, and loss of independence [13]. These psychosocial consequences can further exacerbate the risk of falls by creating a vicious cycle.

Identifying individuals at elevated risk of falling constitutes a critical aspect of preventing falls. An individualized approach to screening, assessment, and intervention is highlighted in professional guidelines, exemplified by practices outlined in the German nursing expert standards for fall and fracture prevention [14] and in the World guidelines for fall prevention for older adults [1]. Although no unanimous agreement exists on the precise selection of fall risk assessment methods [15], an emphasis on an individualized, multifactorial, and comprehensive assessment of fall risk is consistent across professional guidelines [1,14], allowing for the development of tailored multifactorial measures to address fall risk. In accordance with these guidelines, a comprehensive assessment should encompass various domains, including mobility, sensory function, activities of daily living, cognitive function, autonomic function, disease history, medication history, nutrition history, and environmental risk. This personalized approach can enable the efficient identification of older adults at risk of falling, facilitating the implementation of targeted interventions to mitigate this risk.

At present, methods for predicting falls in older adults mostly depend on single assessments [15]. These assessments, being singular tools, may lack the scalability and real-time capabilities necessary for widespread multifactorial fall prevention efforts [1,15]. To embrace a multifactorial approach in fall prevention, the incorporation of technology, such as wearables and mobile health (mHealth) technologies, could prove beneficial. These technological tools possess the capability to capture diverse data types, thereby offering the potential for implementing a comprehensive strategy for multifactorial fall prevention. Some mHealth tools have been developed to implement multifactorial fall risk assessments and have focused on validating both their mobile technology (eg, validation of their inertial measurement units against gold standard technologies) and their application in real-world environments (eg, retirement communities) [16]. Most published research related to fall risk assessments has focused on the ability to discriminate between fallers and nonfallers, determining cutoffs, and assessing their sensitivity and specificity [16,17]. Although this is relevant for identifying individuals at an increased risk of falls, the provision of minimal clinically important differences (MCIDs) is missing [16-21]. Such metrics are crucial for identifying responsiveness to fall prevention programs in effectiveness trials using multifactorial risk assessments as a measure of fall risk.

Many of the available mHealth tools use a combination of functional assessments and questionnaires to gather additional risk factors as part of the multifactorial approach to fall risk assessment [16]. For instance, the Steady app incorporates a health history questionnaire alongside a progressive postural stability test, which informs a weighted algorithm to determine the fall risk [22,23]. Similarly, the Kinesis Balance app enables measurements of standing balance, supplemented by a questionnaire addressing further fall risk factors [24]. Another app, the Aachen Falls Prevention App, is based on the Aachen Falls Prevention Scale and evaluates fall risk through a series of questionnaires and a single balance task [25]. The app developed by Taheri-Kharameh and colleagues assesses fall risk based on the Stopping Elderly Accidents, Deaths, and Injuries framework and incorporates a Timed Up and Go test to categorize individuals regarding their fall risk [26]. Most available apps primarily focus on balance assessments and do not incorporate gait-related information, which is relevant for understanding fall risk [15,27]. Although some apps include a Timed Up and Go test [26,28], it focuses solely on the time taken to complete the test, lacking insight into gait parameters. For instance, among other factors, step length, gait speed, and dynamic trunk sway [29-31] have been identified as parameters contributing to fall risk. To the best of our knowledge, the LINDERA Mobility Analysis (LINDERA GmbH) mHealth app uniquely incorporates gait parameters derived from the smartphone camera, and also includes a questionnaire addressing supplementary fall risk factors, providing a multifactorial approach for fall risk assessment.

The LINDERA Mobility Analysis mHealth app comprehensively captures intrinsic (such as comorbidities, incontinence, fear of falling, and prior falls) and extrinsic (including mobility aids, environmental barriers, and home footwear) factors. These factors, recognized as contributors to the risk of falling [1], are seamlessly integrated into the mHealth app’s calculation of the Fall Risk Score (FRS). The FRS has been previously detailed and evaluated to ascertain its discriminatory efficacy in distinguishing between fallers and nonfallers within a cross-sectional study design [32]. The FRS exhibits performance metrics that are comparable to those of established assessments commonly used for the evaluation of fall risk [32]. Through this, the mHealth app can provide personalized assessments of fall risk and recommendations for fall prevention [32,33]. However, the prognostic value of the FRS has yet to be reported. This study aimed to investigate the prognostic validity of the FRS based on longitudinal real-world data. It is hypothesized that the FRS serves as a prognostic indicator, suggesting an association with future falls within a predetermined time interval. As a secondary goal, this research aims to establish the clinical relevance of the FRS by identifying threshold values and the MCIDs.

## Methods

### Study Design

This is an observational, retrospective cohort study in older adults undergoing fall prevention assessment.

### Setting

This study focused on nursing facilities for older adults within Germany. These institutions, tailored to the distinctive needs of older adults, adhere to routine fall prevention practices mandated by German legislation. Furthermore, these facilities have incorporated LINDERA into their fall prevention assessment strategy, conducting fall prevention assessments approximately every 3 months, contingent upon the absence of any reported falls.

### Participants

Data acquisition involved retrieving data in August 2023 from the LINDERA database. Prior to participant data selection, precise criteria were delineated to identify and incorporate the data relevant to the investigation.

Inclusion criteria were defined as follows: (1) participants should have completed more than one fall risk prevention assessment and (2) participants should be aged 60 years or older. Assessments were exclusively accepted if captured through a uniform app version (versions 10.0.6 to 10.17.0), ensuring consistency in the used FRS calculation, questionnaire version, and gait parameters calculation. Additionally, assessments were considered if they exhibited an interval between 45 and 180 days, corresponding to an initial assessment (T1) and a subsequent follow-up assessment (T2). Repeated assessments (observations) for the same individual were also considered. As the app involved a video-based fall risk assessment, all participants were required to be capable of walking at least 6 meters.

After data retrieval, assessments were included in the analysis only if the gait videos showed the presence of older adults, meaning real human measurements had been collected. To guarantee a dependable documentation of fall history, the analysis focused solely on residents from residential care settings. The study’s final sample size comprised 617 older adults (857 observations), with data collected between June 2021 and July 2023.

### Description of mHealth App

**Figure 1.**
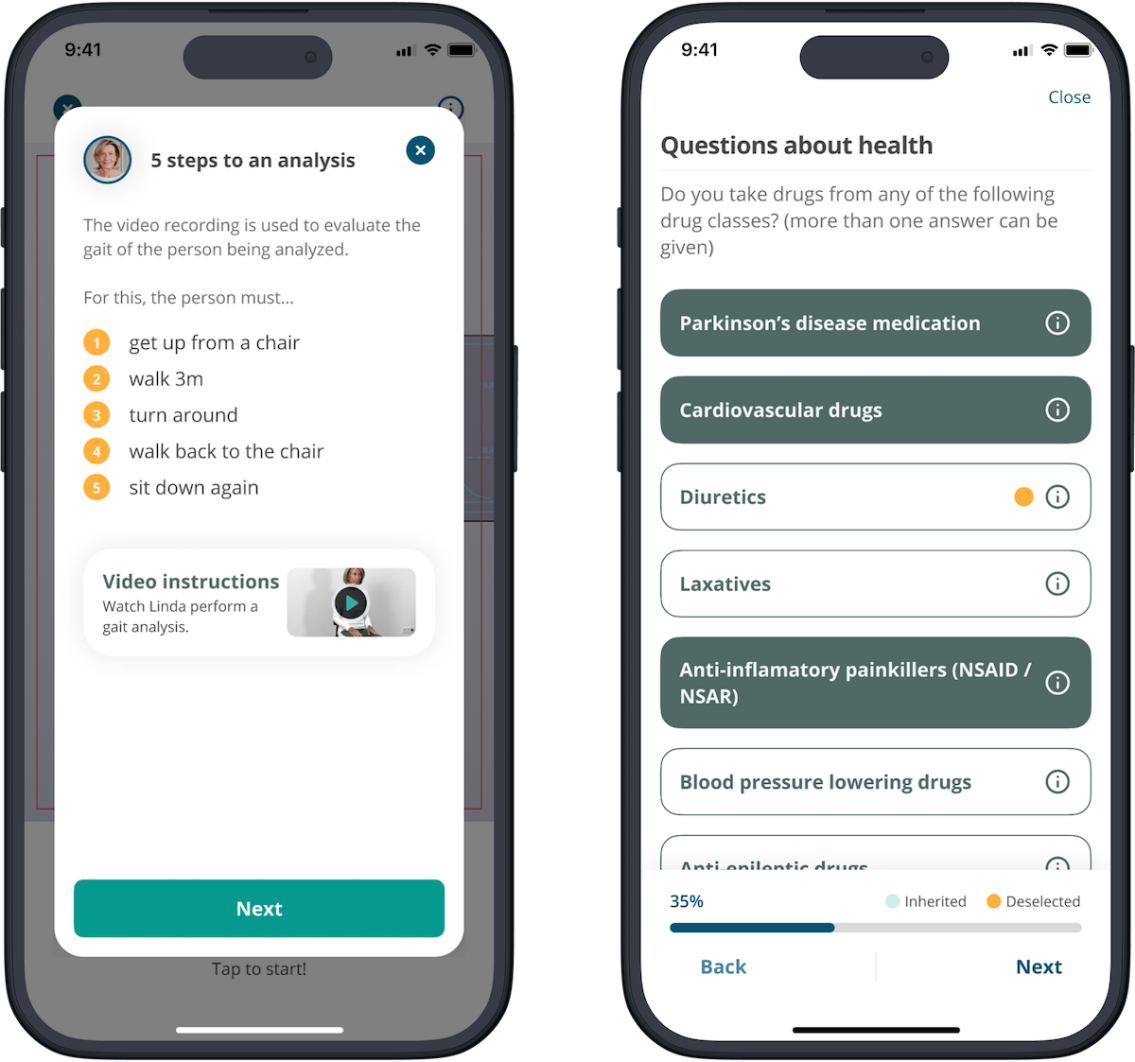
Screenshots depicting the mobile health app during the initialization phase of a new fall prevention assessment.

The mHealth app (English version: Figure 1; German version: Multimedia Appendix 1) was used for the evaluation of fall risk among the study participants through the computation of an FRS, as described elsewhere [32]. Briefly, to complete a fall risk assessment, two procedures are required: (1) recording the participants’ gait via smartphone-based video to evaluate the gait pattern through a computer vision algorithm [34] and (2) the collection of additional fall risk factors through a standardized questionnaire integrated within the mHealth app.

For the video procedure, participants were required to perform a 3-meter walking test involving the sequence of standing up from a chair, walking approximately 3 meters toward the camera, executing a 180-degree turn, returning to the starting point, and sitting down. Participants were permitted to utilize assistive mobility devices (eg, a cane, crutch, or walker) during the video capture process. The video was recorded by a caregiver, such as a nurse, who simultaneously ensured patient safety. The underlying computer vision algorithm is based on a modular system consisting of a video tester, a skeleton estimator (skeleton estimator 2D, skeleton estimator 3D, skeleton optimization 3D), and processing of mobility parameters [34]. The modular artificial intelligence–based algorithm detects gait cycles during the walking segment of the assessment and computes gait parameters such as step length and gait speed. The accuracy of this algorithm has been previously evaluated through comparison with the GAITRite walkway system elsewhere [34], with reported intraclass correlation coefficient values exceeding 0.90, indicating excellent agreement, as detailed in prior research. Following video capture, immediate quality checks are conducted to confirm that the video complies with the predefined quality criteria (eg, exposure, camera movement). If necessary, a new video recording is requested.

Following the video procedure, participants were required to complete a questionnaire comprised of a maximum of 58 questions that encompassed person-related risk factors and environmental risk factors. Participants had the option to fill out the questionnaire either independently or, if they preferred, with the help of caregivers. Additionally, if cognitive limitations prevent a participant from completing the questionnaire, an alternative version is triggered for external assessment by the caregiver. Nevertheless, the same risk factors are collected.

Assessments were analyzed only if the 2 procedures were carried out and uploaded.

### Variables Extracted From the mHealth App

#### Fall Risk Score

Utilizing these 2 procedures (video and questionnaire) inherent to the mHealth app, fall risk factors were systematically and automatically gathered. The recorded video exclusively allows an analysis of the gait pattern, which includes key characteristics such as step length and gait speed. The questionnaire screens for a range of additional fall risk factors, including dizziness and visual and acoustic impairments, among others, which are relevant for a comprehensive assessment of fall risk [32]. There are no overlaps between the risk factors derived from the video and those obtained from the questionnaire; each risk factor is exclusively captured from 1 of these 2 sources.

Each risk factor identified through these methods is considered in the FRS calculation, which is quantified on a scale ranging from 0-100 points. A score of 0 indicates the absence of fall risk factors, while a score of 100 represents the complete presence of all identified fall risk factors. The higher the FRS, the more fall risk factors are present. According to established fall-risk models with demonstrated diagnostic accuracy [35,36], such as St. Thomas’s Risk Assessment Tool in Falling Elderly Inpatients, the Hendrich II Fall Risk Model, and the Downton Fall Risk Assessment, 9 specific risk factors are assigned double weighting. These factors include limited mobility, dizziness, visual and auditory impairments, medication use, cognitive impairment, depression, urge incontinence, a history of falls, and restlessness. Additional risk factors are assigned single weighting. These include comorbidities that limit mobility, foot disorders, conditions that can cause syncope, fear of falling, the use of walking aids, and environmental hazards.

#### Number of Falls at Follow-Up Assessment

The number of falls recorded at follow-up, T2, was self-reported by the senior using the mHealth app. Participants responded to a specific inquiry presented in the mHealth app, detailing how often falls occurred since the last analysis. It is noteworthy to highlight that, owing to the nature of the setting being stationary residential, the number of falls is meticulously documented in this environment. Caregivers have ready access to this documented information when conducting the fall risk prevention assessment.

To standardize these reported values and account for variable observation periods, a normalization process was implemented where the number of reported falls was divided by the corresponding number of days covered by the analysis (T1 to T2).

### Bias

To address potential biases inherent in the study design [37] that could potentially introduce confusion and affect the precision of association estimates, a series of methodological strategies were implemented to enhance the internal validity of the study findings.

### Statistical Analysis

#### Overview

Descriptive statistics were calculated as mean and standard deviation for continuous data and counts for categorical data. For all statistical evaluations, the significance level was set at *α*=.05. RStudio (R version 4.3.1; Posit PBC, irr package) and Python (version 3.9; Python Software Foundation) were used to process, analyze, and visualize all data. Specifically for Python, NumPy (version 1.25.2), Pandas (version 2.03), and scikit-learn (version 1.3.0) libraries were used.

#### Regression Analysis

To determine the relationship between the FRS at T1 and the number of falls at T2, a correlation analysis was performed, with a 3-step approach. First, to enhance data smoothing and establish a more consistent data representation, a running average was computed. This running average utilized the FRS as a grouping parameter, with a window size equivalent to 2% of the FRS value, resulting in the derivation of an average number of falls at T2 for each FRS. Second, multiple regression models were created to identify the function that best fits the data points. To assess the performance of the fitted function, root mean square error (RMSE) was reported. The model exhibiting the lowest RMSE value was selected as the most appropriate. Finally, in the third step, the Spearman rank correlation coefficient was used to evaluate the strength and direction of the monotonic relationships between FRS at T1 and number of falls at T2, both extracted from the running average. This nonparametric test was selected for its ability to handle ordinal data and nonlinear relationships. Correlation coefficients and corresponding *P* values were computed to assess the statistical significance of these associations. This analysis was also conducted in multiple subgroups to identify stronger associations within specific variables. Specifically, the following variables were investigated: time interval (60, 90, or 120 days); number of reported diseases (cancer, arthritis, osteoarthritis, stroke, Parkinson disease, multiple sclerosis, chronic pain, heart disease, lung disease, kidney disease, liver disease, HIV, osteoporosis, dementia, anemia); age (<65, 65-74, 74-85, or >85 years old); gait speed (above or below 0.6 m/s); dementia (with or without); gait speed and dementia (above 0.6 m/s and without dementia, below 0.6 m/s and with dementia); fall history (yes or no); and use of walking aids (yes or no). The subgroups were selected based on risk factors previously mentioned as associated with increased risk of a fall [1,15,38-43]. For each subgroup explored, the RMSE, Spearman correlation coefficients, *P* values, and sample size are reported.

#### Threshold Values

Following this process, threshold values were determined based on the obtained regression model for the main sample size. The corresponding model’s equation was used to estimate the average number of falls per week. To estimate the threshold values over longer periods—such as 6, 12, and 24 months—the average number of falls per week was multiplied by the corresponding number of weeks in those periods (26, 52, and 104, respectively). This approach enabled the calculation of fall risk thresholds over extended time frames.

#### Determination of MCID

To determine the MCID for the FRS, a distribution-based approach was used, in accordance with [44]. Consequently, the distribution-based MCID was computed using the following formula: effect size × SD pooled, where the effect size assumes values of 0.2, 0.5, and 0.8, representing small, medium, and large effect sizes, respectively, in accordance with [45]. The pooled standard deviation (SDpooled) is determined as the square root of [(SD baseline)^2^ + (SD follow-up)^2^/ 2] in [44,46]. The 95% CIs were estimated using bootstrapping methodology. The MCID range was estimated by considering values that span from smaller to larger, as derived from these calculations.

### Ethical Considerations

Data acquisition involved retrieving data in August 2023 from the LINDERA database, encompassing participants who had provided informed consent for the collection and utilization of their data for research purposes. Participants were not compensated for their participation. LINDERA adheres to the European Union General Data Protection Regulation, and all data incorporated into the study were pseudonymized. The study was approved by the local ethics committee at Charité - Universitätsmedizin Berlin (EA4/009/21).

## Results

### Participants

**Figure 2.**
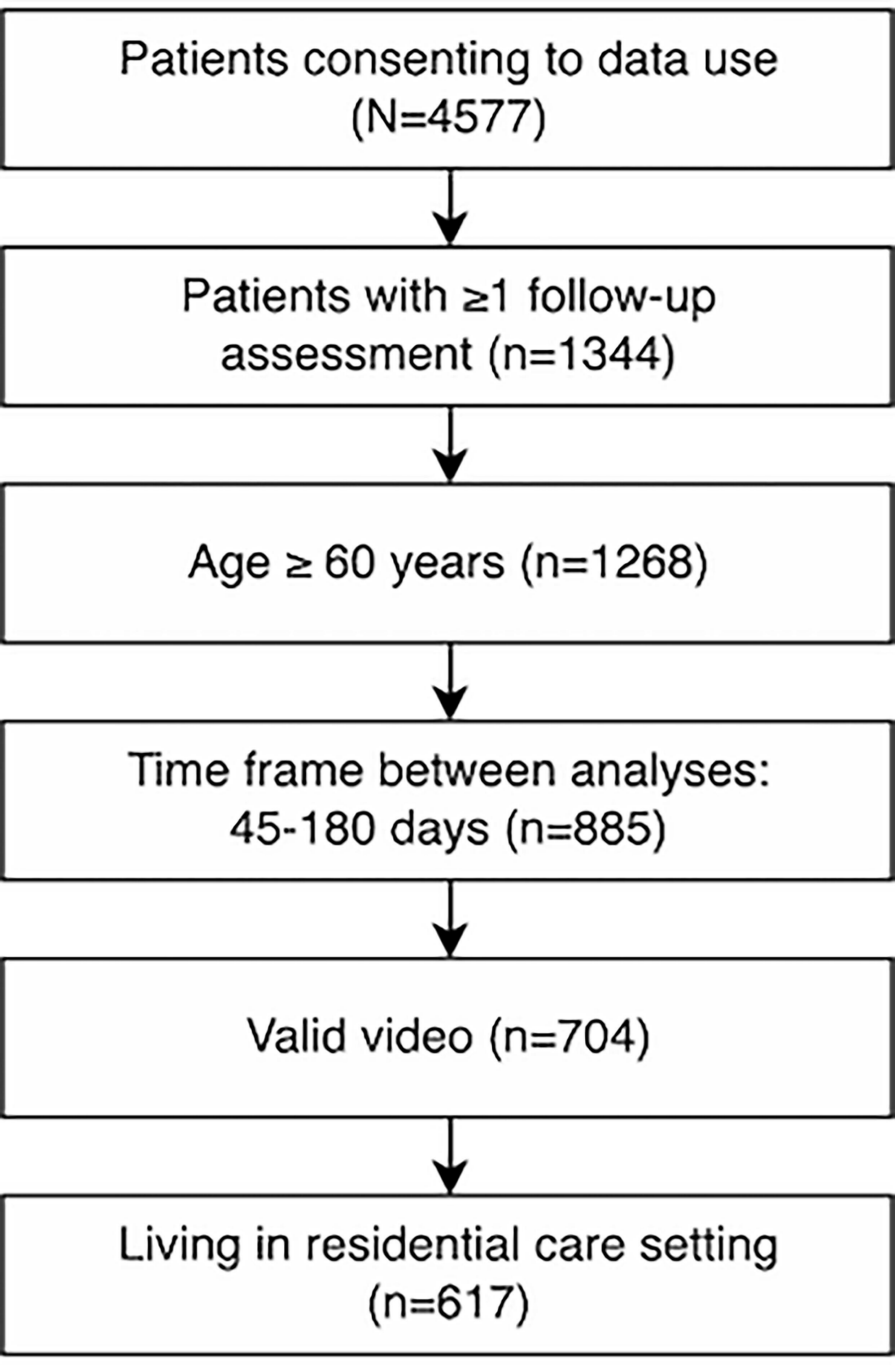
Participant inclusion flowchart.

A detailed participant inclusion flowchart before exclusion is provided in Figure 2, illustrating the selection process. Initially, 4577 patients (3146 women and 1431 men) who provided consent to use their data for research purposes were considered for inclusion. The average age of these participants was 81.1 (SD 12.1) years. Among them, 1192 (25.8%) had dementia and 3235 (70.7%) used walking aids.

The details of the full sample at T1 are displayed in Table 1. The FRS for the observations of the full sample size was 29.7 (SD 11.8%; range 1‐71) at T1 and 29.2 (SD 11.5%; range 4‐77) at T2. The average time interval between T1 and T2 was 108.5 (SD 31.6) days.

At T1, a total of 190 patients (with a total of 277 observations) could not self-report the fall risks collected through the questionnaire and required an external assessment. Comparisons between this group and the self-reported group showed no statistically significant differences in gender (*P*=.31) and fallers at T1 (*P*=.40). However, statistically significant differences were found for age (*P*<.001), gait speed (*P*=.03), and walking aid usage (*P*<.001). Specifically, the self-reported assessment group was older and slower and had a higher proportion of individuals using walking aids (464/580 observations, 80%) compared to the external assessment group (158/277 observations, 57%).

**Table 1.** Demographic and anthropometric data at initial assessment (T1) for the full sample, the self-reported fall risk questionnaire sample, and the externally assessed fall risk questionnaire sample.

Initial assessment characteristic	Full sample	Self-reported questionnaire	Externally assessed questionnaire
Participants, n	617	427	190
Observations, n	857	580	277
Gender, n	615 women, 242 men	423 women, 157 men	192 women, 85 men
Age (years), mean (SD)	83.3 (8.7)	84.0 (8.8)	81.8 (8.4)
Dementia, n	277	0	277
Fallers, n	125	80	45
Gait speed (m/s), mean (SD)	0.49 (0.19)	0.48 (0.19)	0.52 (0.20)
Using walking aids, n	622	464	158

### Regression Analysis

**Figure 3.**
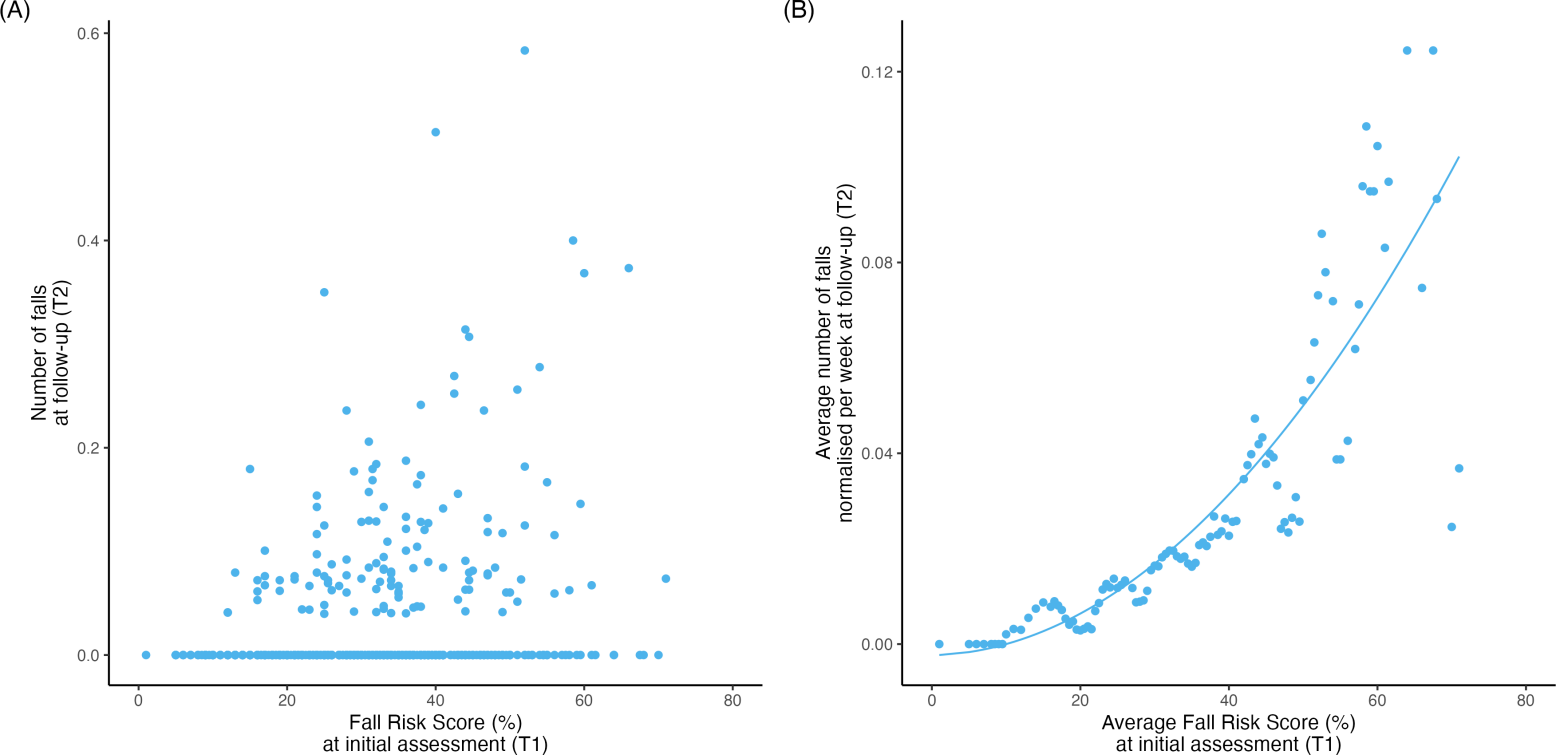
(A) Scatter plot of the FRS at T1 and normalized number of falls per week at T2. (B) Resultant values for the running average computation for the FRS at T1 and normalized number of falls per week at T2, including of the quadratic model in the resultant values of the running average computation. FRS: Fall Risk Score.

Figure 3A presents a scatter plot illustrating the relationship between the normalized number of falls at T2 and the FRS at T1. The mean (SD) normalized number of falls per week at T2 for the full sample was 0.02 (SD 0.05). A running average was calculated using the FRS as a grouping parameter, with a window size equivalent to 2% of the FRS value. Figure 3B displays the resultant points of this running average computation, revealing a nonlinear relationship in the data.

Multiple regression models were computed to determine the best-fitting function. Three sequential models were tested: linear, quadratic, and exponential, introduced in ascending order of complexity. Among the models, the quadratic model (Figure 3B) was selected as it exhibited the lowest RMSE (0.015). The corresponding quadratic regression model’s equation is as follows, where FRS represents the average Fall Risk Score at T1:


$$average\;number\;of\;future\;falls\;per\;week=\;2.02977245\times 10^{-5}\times FRS^{2}+3.1456122\times 10^{-5}\times FRS-2.34295251\times 10^{-3}$$

The linear and exponential models exhibited corresponding RMSE values of 0.017 and 0.016, respectively.

Spearman correlation analysis, using the moving average data as the basis, revealed a strong, positive correlation between FRS at T1 and falls at T2 (ρ=0.960, *P*<.001). Subgroup analysis, using the moving average as the basis, was conducted to explore the impact of various variables on the relationship between FRS at T1 and falls at T2. The outcomes of the subgroup analysis are summarized in Table 2.

Table 2 reveals that significant strong correlations were found for the subgroup using walking aids (ρ=0.955, *P*<.001), with a gait speed below 0.6 m/s (ρ=0.954, *P*<.001), and with a time interval of 120 days (ρ=0.934, *P*<.001), indicating robust associations. Moderate yet significant correlations were observed for the subgroup aged 65‐74 years (ρ=0.437, *P*=.002) and with no diseases (ρ=0.504, *P*=.002). Weak and nonsignificant correlations were found for groups such as those with 3 diseases and gait speed above 0.6 m/s, suggesting no substantial relationship. The results obtained using the raw data are presented in Multimedia Appendix 2.

**Table 2.** Performance metrics of the models evaluated (running average–based; root mean square error) and Spearman correlation analysis to predict number of falls at T2 based on Fall Risk Score values at T1 for subgroups.^a^^,^^b^

Subgroups explored at T1		Root mean square error	Spearman correlation coefficient	*P* value	Fall Risk Score at T1, mean (SD)	Observations, n
**Time interval between T1 and T2**						
	60 days	0.08	0.739	<.001	28.5 (13.7)	36
90 days	0.03	0.833	<.001	30.6 (12.9)	268
120 days	0.02	0.934	<.001	30.0 (12.1)	611
**Diseases, n**						
	0	0.01	0.504	<.001	21.0 (9.9)	107
1	0.05	0.807	<.001	26.2 (11.1)	221
2	0.04	0.800	<.001	29.7 (11.1)	223
3	0.03	0.166	.19	32.8 (10.5)	167
≥4	0.02	0.719	<.001	37.9 (10.4)	139
**Age (years)**						
	<65	0.03	0.167	.38	24.4 (9.4)	47
65‐74	0.03	0.437	.002	27.4 (12.0)	93
74‐85	0.03	0.778	<.001	29.2 (12.4)	324
>85	0.02	0.760	<.001	31.2 (11.3)	393
**Gait speed (m/s)**						
	≥0.6	0.02	0.130	.32	21.9 (9.5)	215
<0.6	0.02	0.954	<.001	32.3 (11.4)	642
**Dementia**						
	Yes	0.04	0.719	<.001	29.2 (11.1)	242
No	0.02	0.748	<.001	29.8 (12.1)	615
**Gait speed (m/s) and dementia**						
	≥0.6 and without dementia	0.01	0.019	.90	21.9 (10.3)	140
<0.6 and with dementia	0.04	0.666	<.001	32.4 (10.8)	167
**Fall history**						
	Yes	0.05	0.729	<.001	40.2 (12.0)	125
No	0.02	0.571	<.001	29.7 (11.8)	857
**Use of walking aids**						
	Yes	0.02	0.955	<.001	32.4 (11.3)	622
No	0.03	0.664	<.001	22.3 (9.8)	235

a T1: initial assessment.

b T2: follow-up assessment.

### Threshold Values

The threshold values obtained from the quadratic model revealed the corresponding FRS values as follows: an expectation of 1 fall within the next 6 months aligns with an FRS value starting at 45%, while an anticipation of 1 fall within the next 12 months corresponds to an FRS value starting at 32%. Additionally, individuals expecting 1 fall in the upcoming 24 months exhibited an associated FRS value starting at 24%.

### MCID Values

Table 3 reports the distribution-based MCID (95% CI) values for the FRS change from T1 to T2.

**Table 3.** Distribution-based MCID values for the Fall Risk Score change from T1 to T2.^a^^,^ ^b^^,^ ^c^

Effect size	Distribution-based MCID (95% CI) for Fall Risk Score
Small (effect size=0.2)	2.3 (2.2-2.4)
Medium (effect size=0.5)	5.8 (5.6-6.1)
Large (effect size=0.8)	9.3 (8.9-9.8)

a MCID: minimal clinically important difference.

b T1: initial assessment.

c T2: follow-up assessment.

## Discussion

### Principal Findings

The study confirms the multifaceted nature of fall risk for older adults from several long-term care facilities (inpatient caregiving) as represented by the FRS. The main findings highlight the prognostic significance of the FRS across different time intervals. Individuals with an FRS exceeding 45% had an increased risk of falling within 6 months, emphasizing the importance of early intervention for those identified at this threshold. The graded risk spectrum, with FRS values of 32% and 24% corresponding to expected falls over 12 and 24 months, respectively, provides a nuanced framework for risk stratification and tailoring of preventive strategies over different time horizons. Additionally, establishing MCID values for FRS offers a robust metric to evaluate the clinical impact of FRS measurement and its corresponding fall prevention programs. By providing MCIDs for small (effect size=0.2), medium (effect size=0.5), and large (effect size=0.8) effect sizes, a framework for interpreting the observed changes in fall risk scores was established, which will help in understanding the results of future prospective studies by evaluating whether observed changes in fall risk scores are meaningful.

Subgroup analyses broaden the understanding of the FRS’s contextual applicability, revealing that the use of walking aids, slower gait speed, and the time interval between assessments are important contributing factors influencing fall risk. In contrast, faster gait speeds and a combination of faster gait speeds and no dementia (indicating higher functional levels) do not appear to be significant contributing factors to fall risk. These insights into FRS dynamics underscore the need for a multifactorial approach to fall risk assessment and management, incorporating clinical, behavioral, and environmental factors for optimal outcomes for people exposed to fall risks.

Overall, the study aimed to assess the prognostic validity of the FRS based on longitudinal fall risk assessment data acquired in real-world conditions. The study hypothesis posited that the FRS operates as a prognostic indicator, suggesting an association with future falls within a predetermined time interval. The study’s main finding was that the FRS, when assessed at T1 and used in a quadratic model to predict the number of falls at T2, demonstrates a good predictive performance. This is supported by the significant strong correlations detected between the FRS at T1 and falls at T2 (ρ=0.960, *P*<.001).

### Prognostic Validity of the Fall Risk Score

The best-fitting regression model, characterized by the lowest RMSE, depicted a nonlinear relationship between the FRS and number of falls. These results seemed to be consistent with previous research. Notably, alternative models for predicting falls rely on objective metrics such as gait speed. Quach and colleagues [47] identified a nonlinear, U-shaped relationship between gait speed and the frequency of falls in community-dwelling older adults. Both faster and slower gait speeds were associated with the highest risk of falls. Subsequent subgroup analyses in this investigation revealed that variations in gait speed may influence the magnitude of the association between FRS and fall frequency, as significant, strong correlations were detected for slower walkers (gait speed below 0.6 m/s), in agreement with findings from [47]. Moreover, the mean FRS values of slower walkers (mean 32.3, SD 11.4%) exceeded the threshold indicative of distinguishing fallers from nonfallers (27.5%), as previously determined [32]. In contrast, the mean FRS values of faster walkers (mean 21.9, SD 9.5%) did not surpass this threshold, underscoring the relevance of gait speed in the predictive model.

Spearman correlation analysis revealed differing strengths of correlation across different subgroups. It was most effective for predicting falls among older individuals with slower gait speeds, using walking aids, and with a time interval between fall risk assessments of 120 days. On the other hand, the correlations were weaker among individuals with different disease counts and those with a combination of gait speed and dementia status. These results indicate that, while the predictive model is robust in certain contexts, it may require additional refinement or consideration of other factors to improve its predictive power across all subgroups. For instance, in the disease group with 3 conditions (n=3), the observed low Spearman correlation can be attributed to the presence of observations with high FRS and zero falls (Multimedia Appendix 3). These observations disrupt the rank-order relationship, leading to a reduction in the Spearman correlation coefficient. Nonetheless, the low RMSE indicates that the quadratic model performed well overall for most data points within this subgroup. Individuals with multiple diseases exhibited a descriptively higher fall risk (Table 2), with an even greater fall risk observed in those over 85 years of age (Multimedia Appendix 4). Health care professionals may be more vigilant in monitoring these individuals, paying closer attention to their health conditions, environmental hazards, and behaviors that could contribute to falls. Such heightened awareness has been previously reported to influence their decisions and actions to prevent falls [48], which may help explain the lack of a statistically significant association between the variables in this subgroup.

An increasing number of mHealth solutions are contributing to the growing body of evidence that supports the validity of using this technology for fall risk screening [49]. For example, the Aachen Falls Prevention Scale, a self-assessment tool that combines a short questionnaire with a balance test, has been found to significantly correlate with users’ self-reported history of falls [25]. In addition, Ozinga and Alberts [50] evaluated postural stability assessments captured by a tablet, comparing their results to those obtained through a 3D motion analysis system. Their findings indicate that tablet sensors can quantify postural stability with sufficient accuracy. A more recent review also evaluated several digital apps developed for fall risk assessment [16]. The accumulating body of evidence supporting mHealth use for assessing fall risk holds significant potential to optimize fall prevention efforts by not only identifying individuals at high risk but also delivering targeted interventions tailored to the specific needs identified. This opens avenues for older adults to assess their individual fall risk, a crucial step in determining the appropriate type of fall prevention treatment. However, the majority of mHealth tools assessed in the literature only provide support and integration with isolated parts of fall risk assessment and fail to incorporate a multifactorial, multimodal approach.

### MCID Findings

The results from the quadratic model established threshold values for the FRS that correlated with the expectation of falls over different time frames, resulting in risk stratification. The calculation of MCID for FRS changes from T1 to T2, with varying effect sizes, enabled clinicians to understand the magnitude of change in FRS that would be considered clinically significant. Subgroup analysis further refined the understanding of how various characteristics influenced the FRS and the implementation of recommendations, highlighting the complex interplay of factors such as disease presence, age, gait speed, dementia status, fall history, and the use of walking aids in fall risk and response to interventions. These findings underscore the importance of personalized approaches in managing fall risk among different populations, which was not part of the real-world usage of the device.

The distribution-based approach allowed for the interpretation of the MCID while taking into consideration its inherent variability [51]. This method informed the estimation of the MCID by incorporating descriptive statistics derived from the observed scores across the sample’s distribution [52]. The magnitude of effect sizes varied with the clinical condition under consideration, with small effect sizes holding potential significance for patients with severe disease, while moderate to large effect sizes become relevant for patients with milder conditions [44]. Among distribution-based methods, the standard deviation between participants is one of the most widely accepted [51,53], and both the variability at initial and follow-up assessments should be considered [46].

Distribution-based approaches are validated methods to derive MCIDs [53,54]. These studies support the use of distribution-based MCID methods, demonstrating accuracy and reliability in deriving MCIDs. In this study, distribution-based methods to derive MCIDs from real world baseline data were effectively applied. In the results, the MCID was divided into 3 categories: small change of approximately 2.3 points, medium change of approximately 5.8 points, and large change of approximately 9.3 points. These MCID numbers can be used to evaluate whether the changes in FRS after an intervention (eg, an exercise program) are large enough to be meaningful. If a participant’s FRS drops by more than the MCID, it is likely that the program was beneficial. As a result, the distribution-based approach yielded appropriate differences for a meaningful clinical interpretation of FRS changes.

### Usage of Real-World Data

Regulatory organizations are progressively acknowledging the utility of real-world data (RWD) in forming real-world evidence. A systematic literature review was recently undertaken to examine the use of RWD for interpreting outcomes from trials lacking control groups [55]. The study examined major regulatory and health technology assessment bodies and underscored the necessity for enhanced guidance on methodological considerations from these bodies. The systematic review articulated essential directives for generating real-world evidence that is appropriate for its intended benefits. The application of RWD is particularly pertinent in areas where randomized clinical trials are not viable, such as in oncology, rare diseases, or nursing homes [56,57]. Here, controls may be derived from historical data and observational studies. Finally, the paper advocated for the exchange of experiences among stakeholders (eg, sponsors and regulatory bodies) to promote learning and refine the application of RWD-derived evidence, aiming to enhance patient care.

### Limitations

#### Study Group

This study included the physical and demographic characteristics of older adults, providing valuable insights for aging populations. However, its focus on older individuals, gender imbalance, and lack of specific details regarding dementia and fall circumstances could limit its generalizability. Notably, there are indications that gender could have a significant influence on fall risk factors [58] and fall awareness behavior should be emphasized among older females to address gender-specific factors that might be crucial in mitigating fall risk in this demographic [59]. Furthermore, variable assessment intervals may affect the reliability of longitudinal comparisons.

#### Study Type

The study relies on preexisting, retrospective data, which may have limitations in accuracy, completeness, and consistency. There is also a risk of selection bias, as the data might not have been collected with the current research questions in mind (eg, without a control group, it is harder to attribute changes in fall risk solely to the digital interventions studied). This limits the ability to establish cause-and-effect relationships. Although RWD are valuable for their practical relevance, they can introduce confounding factors and variability.

#### Statistical Methods

The study suggests that, while there were associations between FRS and fall frequency, there were nonlinear relationships, with variable correlations across different subgroups. The prognostic model was most reliable for predicting falls among older individuals with slower gait speeds and those using walking aids. However, its predictive power was less consistent among individuals with different disease counts and those with a combination of gait speed and dementia status. These results indicate that, while the FRS is robust in certain contexts, it may require additional refinement or consideration of other factors to improve its predictive power across all subgroups.

#### Bias Reduction

Specifically, eligibility criteria were preestablished to ensure consistency in participant selection and minimize the impact of selection bias on the study outcomes. Additionally, quality criteria were defined for both video capture and data collection processes. For video capture, to optimize the reliability of gait parameter calculations from video recordings, predetermined quality criteria—encompassing aspects such as exposure and camera movement—were systematically implemented. After video capture, this quality assurance process was executed to verify adherence to the predefined criteria, ensuring the integrity of the captured data. For the data collection process, to guarantee caregivers’ proficiency in utilizing the mHealth app, a regimen of regular and standardized training sessions was administered by the LINDERA Customer Success Team. Finally, to ensure the reliability of the dataset included, a comprehensive review of all videos associated with the analyses was conducted to confirm the presence of older adults in the video recordings.

In summary, the study utilized the FRS as a predictive tool and the MCID for managing individual fall risk. The FRS could screen fall risks among older adults in nursing care, with its real-world application underscoring its practical utility in clinical settings. The MCID enhanced the study’s findings, while translating statistical shifts in FRS into meaningful, patient-centric outcomes. This approach can be used to personalize care plans and also offers an objective measure for evaluating the efficacy of fall prevention strategies. The deduction of MCID sets a practical standard in geriatric care research and practice, emphasizing its value in assessing the effectiveness of interventions in fall risk management.

### Conclusions

This study investigated the complex nature of falls and fall risk, encapsulated by the FRS and its prognostic value. Elevated FRS values corresponded to an increased risk of falls and future falls. This, along with the obtained threshold, offers a stratified approach to risk assessment and the formulation of preventative strategies tailored to risk projections of falls. The deduction of MCID values for FRS changes provides a metric for assessing the clinical significance of interventions across a spectrum of effect sizes. Despite the stated limitations, the study’s insights are a valuable addition to the existing literature on fall risk, suggesting the FRS as a predictive tool that may benefit from further refinement for broader applications in clinical and nursing settings. The fall risk assessment method utilized in this study identified specific fall risk factors for each individual. By targeting the individual fall risk profile with distinct fall risk factors—such as mobility limitations or environmental hazards—this method enables the delivery of tailored and person-centered fall prevention strategies. These strategies are designed to manage and mitigate the fall risks identified by addressing the particular needs and vulnerabilities of each individual based on a holistic, multifactorial fall risk assessment. Future research should focus on evaluating the effectiveness of such fall prevention strategies derived from a comprehensive and multifactorial fall risk assessment among individuals in residential care settings.

## Supplementary material

10.2196/55681 Multimedia Appendix 1 Screenshots depicting the mobile health app (German version) during the initialization phase of a new fall prevention assessment.

10.2196/55681 Multimedia Appendix 2 Spearman correlation analysis to predict number of falls at T2 based on Fall Risk Score values at T1 for subgroups.

10.2196/55681 Multimedia Appendix 3 Scatter plot of resultant values for the running average of the Fall Risk Score at T1 and normalized number of falls per week at T2 for different combinations of number of diseases. The solid line refers to the quadratic model.

10.2196/55681 Multimedia Appendix 4 Spearman correlation analysis to predict number of falls at T2 based on Fall Risk Score values at T1 for subgroups of individuals aged over 85 years.

## Abbreviations

FRS: Fall Risk Score
MCID: minimal clinically important differences
RMSE: root mean square error
RWD: real-world data
T1: initial assessment
T2: follow-up assessment

## References

[1] Montero-Odasso M, van der Velde N, Martin FC (2022). World guidelines for falls prevention and management for older adults: a global initiative. Age Ageing.

[2] Rubenstein LZ (2006). Falls in older people: epidemiology, risk factors and strategies for prevention. Age Ageing.

[3] Masud T, Morris RO (2001). Epidemiology of falls. Age Ageing.

[4] Karinkanta S, Heinonen A, Sievanen H, Uusi-Rasi K, Kannus P (2005). Factors predicting dynamic balance and quality of life in home-dwelling elderly women. Gerontology.

[5] Haagsma JA, Olij BF, Majdan M (2020). Falls in older aged adults in 22 European countries: incidence, mortality and burden of disease from 1990 to 2017. Inj Prev.

[6] (2021). Falls. World Health Organization.

[7] Montero-Odasso MM, Kamkar N, Pieruccini-Faria F (2021). Evaluation of clinical practice guidelines on fall prevention and management for older adults: a systematic review. JAMA Netw Open.

[8] Khow KSF, Visvanathan R (2017). Falls in the aging population. Clin Geriatr Med.

[9] James SL, Lucchesi LR, Bisignano C (2020). The global burden of falls: global, regional and national estimates of morbidity and mortality from the Global Burden of Disease Study 2017. Inj Prev.

[10] Abolhassani F, Moayyeri A, Naghavi M, Soltani A, Larijani B, Shalmani HT (2006). Incidence and characteristics of falls leading to hip fracture in Iranian population. Bone.

[11] Stevens JA, Adekoya N (2001). Brain injury resulting from falls among elderly persons. JAMA.

[12] Haddad YK, Bergen G, Florence CS (2019). Estimating the economic burden related to older adult falls by state. J Public Health Manag Pract.

[13] Schoene D, Heller C, Aung YN, Sieber CC, Kemmler W, Freiberger E (2019). A systematic review on the influence of fear of falling on quality of life in older people: is there a role for falls?. Clin Interv Aging.

[14] Blumenberg P, Büscher A, Krebs M, Stehling H (2022). Expertenstandard Sturzprophylaxe in Der Pflege [Book in German].

[15] Beck Jepsen D, Robinson K, Ogliari G (2022). Predicting falls in older adults: an umbrella review of instruments assessing gait, balance, and functional mobility. BMC Geriatr.

[16] Hsieh KL, Chen L, Sosnoff JJ (2023). Mobile technology for falls prevention in older adults. J Gerontol A Biol Sci Med Sci.

[17] Tiedemann A, Lord SR, Sherrington C (2010). The development and validation of a brief performance-based fall risk assessment tool for use in primary care. J Gerontol A Biol Sci Med Sci.

[18] Lee V, Appiah-Kubi L, Vogrin S, Zanker J, Mitropoulos J (2023). Current cut points of three falls risk assessment tools are inferior to calculated cut points in geriatric evaluation and management units. Muscles.

[19] Hayashi C, Okano T, Toyoda H (2024). Development and validation of a prediction model for falls among older people using community-based data. Osteoporos Int.

[20] Mishra AK, Skubic M, Despins LA (2022). Explainable fall risk prediction in older adults using gait and geriatric assessments. Front Dig Health.

[21] Ong MF, Soh KL, Saimon R, Myint WW, Pawi S, Saidi HI (2023). Falls risk screening tools intended to reduce fall risk among independent community-dwelling older adults: a systematic review. Int J Nurs Pract.

[22] Hsieh KL, Fanning JT, Rogers WA, Wood TA, Sosnoff JJ (2018). A fall risk mHealth app for older adults: development and usability study. JMIR Aging.

[23] Hsieh KL, Fanning JT, Sosnoff JJ (2019). A smartphone fall risk application is valid and reliable in older adults during real-world testing. Gerontechnology.

[24] Greene BR, McManus K, Ader LGM, Caulfield B (2021). Unsupervised assessment of balance and falls risk using a smartphone and machine learning. Sensors (Basel).

[25] Rasche P, Mertens A, Bröhl C (2017). The “Aachen Fall Prevention App” - a smartphone application app for the self-assessment of elderly patients at risk for ground level falls. Pat Saf Surg.

[26] Taheri-Kharameh Z, Malmgren Fänge A, Ekvall Hansson E (2022). Development of a mobile application to screen and manage fall risks in older people. Disabil Rehabil Assist Technol.

[27] Espy DD, Yang F, Bhatt T, Pai YC (2010). Independent influence of gait speed and step length on stability and fall risk. Gait Posture.

[28] Singh DKA, Goh JW, Shaharudin MI, Shahar S (2021). A mobile app (FallSA) to identify fall risk among Malaysian community-dwelling older persons: development and validation study. JMIR mHealth uHealth.

[29] Rodríguez-Molinero A, Herrero-Larrea A, Miñarro A (2019). The spatial parameters of gait and their association with falls, functional decline and death in older adults: a prospective study. Sci Rep.

[30] Lindemann U, Lundin-Olsson L, Hauer K, Wengert M, Becker C, Pfeiffer K (2008). Maximum step length as a potential screening tool for falls in non-disabled older adults living in the community. Aging Clin Exp Res.

[31] Lee SW, Verghese J, Holtzer R, Mahoney JR, Oh-Park M (2014). Trunk sway during walking among older adults: norms and correlation with gait velocity. Gait Posture.

[32] Rabe S, Azhand A, Pommer W, Müller S, Steinert A (2020). Descriptive evaluation and accuracy of a mobile app to assess fall risk in seniors: retrospective case-control study. JMIR Aging.

[33] Strutz N, Brodowski H, Kiselev J, Heimann-Steinert A, Müller-Werdan U (2022). App-based evaluation of older people’s fall risk using the mHealth app Lindera Mobility Analysis: exploratory study. JMIR Aging.

[34] Azhand A, Rabe S, Müller S, Sattler I, Heimann-Steinert A (2021). Algorithm based on one monocular video delivers highly valid and reliable gait parameters. Sci Rep.

[35] da Costa BR, Rutjes AWS, Mendy A, Freund-Heritage R, Vieira ER (2012). Can falls risk prediction tools correctly identify fall-prone elderly rehabilitation inpatients? A systematic review and meta-analysis. PLoS One.

[36] Park SH (2018). Tools for assessing fall risk in the elderly: a systematic review and meta-analysis. Aging Clin Exp Res.

[37] Taur SR (2022). Observational designs for real-world evidence studies. Perspect Clin Res.

[38] Chantanachai T, Sturnieks DL, Lord SR, Payne N, Webster L, Taylor ME (2021). Risk factors for falls in older people with cognitive impairment living in the community: systematic review and meta-analysis. Ageing Res Rev.

[39] Schoene D, Wu SMS, Mikolaizak AS (2013). Discriminative ability and predictive validity of the timed up and go test in identifying older people who fall: systematic review and meta-analysis. J Am Geriatr Soc.

[40] Mortaza N, Abu Osman NA, Mehdikhani N (2014). Are the spatio-temporal parameters of gait capable of distinguishing a faller from a non-faller elderly?. Eur J Phys Rehabil Med.

[41] Peeters G, Cooper R, Tooth L, van Schoor NM, Kenny RA (2019). A comprehensive assessment of risk factors for falls in middle-aged adults: co-ordinated analyses of cohort studies in four countries. Osteoporos Int.

[42] Jehu DA, Davis JC, Falck RS (2021). Risk factors for recurrent falls in older adults: a systematic review with meta-analysis. Maturitas.

[43] Kearney FC, Harwood RH, Gladman JRF, Lincoln N, Masud T (2013). The relationship between executive function and falls and gait abnormalities in older adults: a systematic review. Dement Geriatr Cogn Disord.

[44] Jehu DA, Davis JC, Madden K, Parmar N, Liu-Ambrose T (2022). Establishing the minimal clinically important difference of the EQ-5D-3L in older adults with a history of falls. Qual Life Res.

[45] Cohen J (1988). Statistical Power Analysis for the Behavioral Sciences.

[46] Lin KC, Hsieh YW, Wu CY, Chen CL, Jang Y, Liu JS (2009). Minimal detectable change and clinically important difference of the Wolf Motor Function Test in stroke patients. Neurorehab Neural Repair.

[47] Quach L, Galica AM, Jones RN (2011). The nonlinear relationship between gait speed and falls: the Maintenance of Balance, Independent Living, Intellect, and Zest in the Elderly of Boston Study. J Am Geriatr Soc.

[48] Takase M (2023). Falls as the result of interplay between nurses, patient and the environment: using text-mining to uncover how and why falls happen. Int J Nurs Sci.

[49] Hamm J, Money AG, Atwal A, Paraskevopoulos I (2016). Fall prevention intervention technologies: a conceptual framework and survey of the state of the art. J Biomed Inform.

[50] Ozinga SJ, Alberts JL (2014). Quantification of postural stability in older adults using mobile technology. Exp Brain Res.

[51] Guyatt GH, Osoba D, Wu AW, Wyrwich KW, Norman GR, Clinical Significance Consensus Meeting Group (2002). Methods to explain the clinical significance of health status measures. Mayo Clin Proc.

[52] Revicki D, Hays RD, Cella D, Sloan J (2008). Recommended methods for determining responsiveness and minimally important differences for patient-reported outcomes. J Clin Epidemiol.

[53] Mouelhi Y, Jouve E, Castelli C, Gentile S (2020). How is the minimal clinically important difference established in health-related quality of life instruments? Review of anchors and methods. Health Qual Life Outcomes.

[54] Watt JA, Veroniki AA, Tricco AC, Straus SE (2021). Using a distribution-based approach and systematic review methods to derive minimum clinically important differences. BMC Med Res Methodol.

[55] Curtis LH, Sola-Morales O, Heidt J (2023). Regulatory and HTA considerations for development of real-world data derived external controls. Clin Pharmacol Ther.

[56] Corrao G, Franchi M, Mancia G (2022). Knocking on heaven’s door: the gap between health institutions and academies in generating knowledge utilizing real-world data. Front Public Health.

[57] Wieseler B, Neyt M, Kaiser T, Hulstaert F, Windeler J (2023). Replacing RCTs with real world data for regulatory decision making: a self-fulfilling prophecy?. BMJ.

[58] Gale CR, Westbury LD, Cooper C, Dennison EM (2018). Risk factors for incident falls in older men and women: the English longitudinal study of ageing. BMC Geriatr.

[59] Goh JW, Singh DKA, Mesbah N, Hanafi AAM, Azwan AF (2021). Fall awareness behaviour and its associated factors among community dwelling older adults. BMC Geriatr.

