# Supplementary material for: Evaluating the Prognostic and Clinical Validity of the Fall Risk Score Derived From an AI-Based mHealth App for Fall Prevention: Retrospective Real-World Data Analysis
Source: JMIR Aging. 2024 Dec 4;7:e55681. doi: 10.2196/55681 (PMC11634047; doi:10.2196/55681)
Supplement: Multimedia Appendix 1 [file aging-v7-e55681-s001.docx]

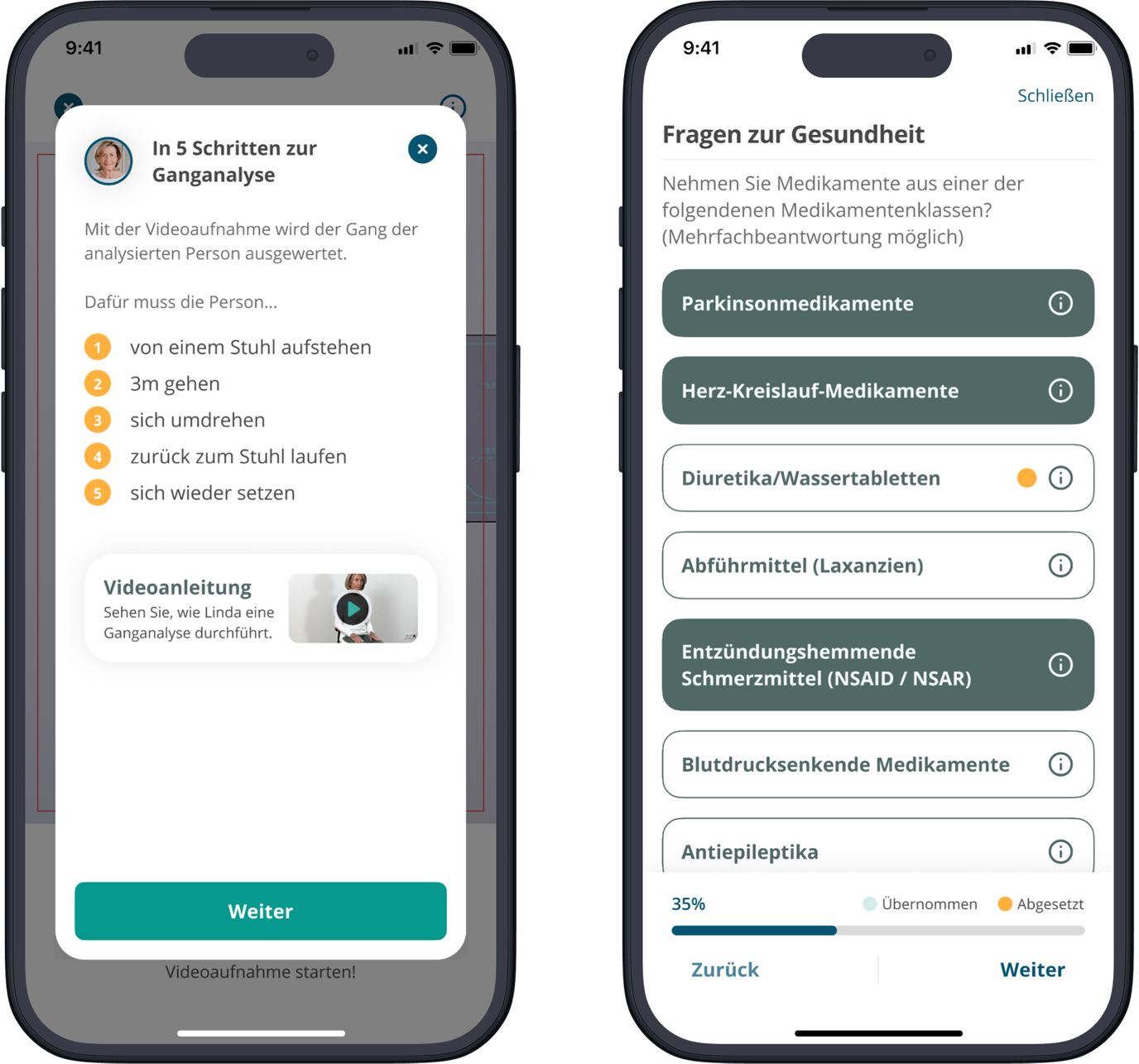


Figure 1. Screenshots depicting the mHealth application (German version) screenshots during the initialization phase of a new fall prevention assessment.
