# Supplementary material for: Evaluating the Prognostic and Clinical Validity of the Fall Risk Score Derived From an AI-Based mHealth App for Fall Prevention: Retrospective Real-World Data Analysis
Source: JMIR Aging. 2024 Dec 4;7:e55681. doi: 10.2196/55681 (PMC11634047; doi:10.2196/55681)
Supplement: Multimedia Appendix 2 [file aging-v7-e55681-s002.docx]

Table S1. Spearman’s correlation analysis to predict number of falls at T2 based on Fall Risk Score values at T1 for sub-groups.^a-f^

| **Sub-groups explored at T1** | | **Spearman's CC** | ***P*** | **Fall Risk Score at T1 (mean** ± **SD)** | **Observations included (n)** |
| --- | --- | --- | --- | --- | --- |
| Complete dataset | | **0.221** | <.001 | 29.7 ± 11.8 | 857 |
| Time interval between T1 and T2 | 60 days | **0.568** | <.001 | 28.5 ± 13.7 | 36 |
|  | 90 days | **0.287** | <.001 | 30.6 ± 12.9 | 268 |
|  | 120 days | **0.234** | <.001 | 30.0 ± 12.1 | 611 |
| Diseases (n) | 0 | **0.240** | .01 | 21.0 ± 9.9 | 107 |
|  | 1 | **0.298** | <.001 | 26.2 ± 11.1 | 221 |
|  | 2 | **0.210** | .002 | 29.7 ± 11.1 | 223 |
|  | 3 | 0.272 | .09 | 32.8 ± 10.5 | 167 |
|  | ≥ 4 | 0.159 | .06 | 37.9 ± 10.4 | 139 |
| Age (years old) | < 65 | **0.292** | .05 | 24.4 ± 9.4 | 47 |
|  | 65 - 74 | 0.202 | .05 | 27.4 ± 12.0 | 93 |
|  | 74 - 85 | **0.259** | <.001 | 29.2 ± 12.4 | 324 |
|  | > 85 | **0.172** | <.001 | 31.2 ± 11.3 | 393 |
| Gait speed (m/s) | ≥ 0.6 | 0.036 | .59 | 21.9 ± 9.5 | 215 |
|  | < 0.6 | **0.223** | <.001 | 32.3 ± 11.4 | 642 |
| Dementia | Yes | **0.251** | <.001 | 29.2 ± 11.1 | 242 |
|  | No | **0.209** | <.001 | 29.8 ± 12.1 | 615 |
| Gait Speed (m/s)  &  Dementia | ≥ 0.6  &  without dementia | 0.041 | .63 | 21.9 ± 10.3 | 140 |
|  | < 0.6  &  with dementia | **0.239** | .002 | 32.4 ±10.8 | 167 |
| Fall history | Yes | **0.237** | .008 | 40.2 ± 12.0 | 125 |
|  | No | **0.118** | .001 | 29.7 ± 11.8 | 857 |
| Use of walking aids | Yes | **0.227** | <.001 | 32.4 ± 11.3 | 622 |
|  | No | **0.188** | .004 | 22.3 ± 9.8 | 235 |

^a^ Spearman’s CC: Spearman’s correlation coefficient

^b^ T1: initial assessment

^c^ T2: follow-up assessment

^d^ m/s: meters per second

^e^ SD: standard deviation

^f^ n: counts
