# Supplementary material for: Evaluating the Prognostic and Clinical Validity of the Fall Risk Score Derived From an AI-Based mHealth App for Fall Prevention: Retrospective Real-World Data Analysis
Source: JMIR Aging. 2024 Dec 4;7:e55681. doi: 10.2196/55681 (PMC11634047; doi:10.2196/55681)
Supplement: Multimedia Appendix 3 [file aging-v7-e55681-s003.docx]

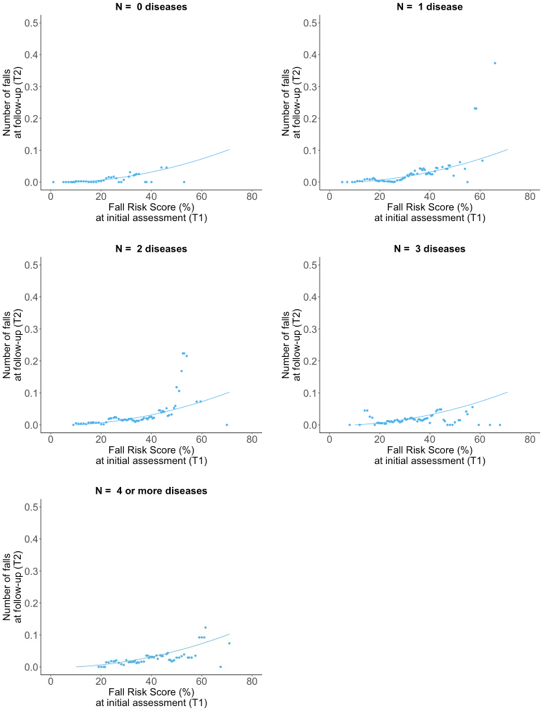


Figure S2 – Scatter plot of resultant values for the running average of the Fall Risk Score (FRS) at T1 and normalized number of falls per week at T2 for different combinations of number of diseases. Solid line refers to the quadratic model.
