# Supplementary material for: Evaluating the Prognostic and Clinical Validity of the Fall Risk Score Derived From an AI-Based mHealth App for Fall Prevention: Retrospective Real-World Data Analysis
Source: JMIR Aging. 2024 Dec 4;7:e55681. doi: 10.2196/55681 (PMC11634047; doi:10.2196/55681)
Supplement: Multimedia Appendix 4 [file aging-v7-e55681-s004.docx]

Table S2. Spearman’s correlation analysis to predict number of falls at T2 based on Fall Risk Score values at T1 for sub-groups of individuals aged over 85 years.^a-f^

| **Sub-groups explored at T1** | | **RMSE** | **Spearman's CC** | ***P*** | **Fall Risk Score at T1 (mean** ± **SD)** | **Observations (n)** |
| --- | --- | --- | --- | --- | --- | --- |
| Time interval between T1 and T2 | 60 days | 0.14 | **0.553** | 0.040 | 25.3 ± 11.1 | 14 |
|  | 90 days | 0.07 | **0.409** | 0.001 | 30.3 ± 12.1 | 114 |
|  | 120 days | 0.03 | **0.648** | <.001 | 31.3 ± 11.7 | 277 |
| Diseases (n) | 0 | 0.01 | **0.610** | <.001 | 21.5 ± 8.7 | 40 |
|  | 1 | 0.04 | **0.584** | <.001 | 27.9 ± 10.6 | 97 |
|  | 2 | 0.05 | **0.447** | 0.001 | 30.8 ± 10.4 | 96 |
|  | 3 | 0.04 | 0.088 | 0.579 | 33.3 ± 9.7 | 84 |
|  | ≥ 4 | 0.03 | 0.076 | 0.626 | 38.5 ± 10.7 | 76 |
| Gait speed (m/s) | ≥ 0.6 | 0.02 | 0.200 | 0.217 | 23.3 ± 10.6 | 73 |
|  | < 0.6 | 0.02 | **0.776** | <.001 | 33.0 ± 10.7 | 320 |
| Dementia | Yes | 0.03 | **0.562** | <.001 | 30.2 ± 10.8 | 99 |
|  | No | 0.03 | **0.517** | <.001 | 31.5 ± 11.4 | 294 |
| Gait Speed (m/s)  &  Dementia | ≥ 0.6  &  without dementia | 0.02 | 0.305 | 0.085 | 24.3 ± 11.3 | 50 |
|  | < 0.6  &  with dementia | 0.03 | **0.469** | 0.002 | 32.8 ± 10.0 | 76 |
| Fall history | Yes | 0.05 | **0.469** | 0.003 | 40.6 ± 11.4 | 62 |
|  | No | 0.02 | **0.380** | <.001 | 29.4 ± 10.4 | 331 |
| Use of walking aids | Yes | 0.03 | **0.666** | <.001 | 32.7 ± 10.9 | 339 |
|  | No | 0.02 | **0.683** | <.001 | 21.8 ± 9.4 | 54 |

^a^ Spearman’s CC: Spearman’s correlation coefficient

^b^ T1: initial assessment

^c^ T2: follow-up assessment

^d^ m/s: meters per second

^e^ SD: standard deviation

^f^ n: counts
